# Chinese institutions should be more proactive and transparent in promoting research integrity: A perspective

**DOI:** 10.3389/frma.2022.999182

**Published:** 2022-11-09

**Authors:** Haihong Zhang

**Affiliations:** The Research Department, Peking University Health Science Center, Beijing, China

**Keywords:** research integrity, institution, compliance, quality, efficiency, policy innovation, experience sharing

## Abstract

China has significantly endeavored to promote research integrity. Institutions, which have been identified as the primarily responsible entity, face challenges and concerns of compliance, quality, and low efficiency. In this perspective, the problems and root causes of these challenging concerns are clarified from the Chinese viewpoint. In conclusion, the opinion that institutions should be more proactive and transparent in promoting research integrity is discussed. A practical suggestion is proposed, including team building, policy innovation, capacity building, researcher empowerment, and experience sharing.

## Introduction

Considerable efforts have been undertaken thus far to promote research integrity in China. Governmental agencies have issued more than 70 research integrity regulations and policies from 1981 to 2020 (Du and Zuo, 2020). According to a quantitative analysis of 20 years of research integrity policies in China, “academic ethics” and “policy reform” were the top two priorities (Sheng et al., 2020). Recent years have witnessed a fast-evolving process of regulatory requirements for research integrity management, research misconduct investigation, ethics review of human subject research, research ethics, and research integrity training. In approximately 30 years of navigation, China has developed institutional level ethics review mechanisms and systems to protect the safety and welfare of human subjects involved in biomedical research. Specifically, a series of significant research integrity regulations were issued in 2018 and afterward. Two of the most influential ones were the *Opinions on Further Strengthening the Development of Scientific Research Integrity* jointly published in 2018 by the General Offices of the Central Committee and the State Council, and the *Rules for the Investigation and Handling of Scientific Research Misconduct Cases* jointly published in 2019 by 20 government departments and agencies, including the Ministry of Science and Technology, National Health Commission, and Ministry of Education.

According to the mandate set forth by current research integrity and research ethics regulations and policies, one of the key strategies adopted to develop a more comprehensive research integrity management system was clarifying the accountabilities and responsibilities of different stakeholders. Institutions including hospitals, academic universities, research institutions, and enterprises were the primarily responsible entities. According to the 2018 Opinion document, institutions must develop institutional-based requirements of research integrity and merge research integrity management into daily work. Establishing an institutional charter and academic committee to handle research integrity issues with a guaranteed budget, office facilities, and full-time personnel, was a mandate, particularly for research institutions and academic universities. The institutional academic committee should be responsible for ensuring research integrity, specifically for the deliberation, assessment, acceptance, investigation, supervision, and consultation of alleged research misconduct. So was the research ethics review requirement. According to the *Guidelines for the Ethics Review of Biomedical Research Involving Human Subjects* (updated in 2016), each institution conducting biomedical research that involves human subjects must establish an institutional ethics review board. Human subject research cannot be initiated without appropriate ethics review approval. On March 20, 2022, the *Opinions on Strengthening the Ethical Governance of Science and Technology* was jointly published by the General Offices of the Central Committee and the State Council, which again set the roadmap and working priorities for research integrity and research ethics in the context of the new era. The administrative responsibilities, the daily working mechanism in research integrity, and the research ethics of institutions were reaffirmed in this iconic document.

Institutions, identified as the primarily responsible entity in the regulatory framework, are expected to play crucial roles in promoting research integrity in China. However, institutions have been facing challenges not only for the practicability of regulatory requirements (Sun and Ren, 2017) but also for the changing landscape of the research paradigm and environment. Ideally, from the policy development perspective, such a design enables different stakeholders to strengthen research integrity as a whole. An increasing number of difficulties and challenges were identified in practice, which became obstacles that adversely affected institutions to fulfill their responsibilities in promoting research integrity. Thus, we must have a timely reflection on institutions' roles, not only from the policy design perspective but also from that of the real-world environment, to examine the possible space for improvement and future sustainable development of research integrity in China. This perspective takes a novel lens to refresh the challenges and possible working strategies for Chinese institutions to enforce research integrity.

## Identified concerns and challenges for institutions in promoting research integrity

First, compliance is identified as the main challenge. In practice, institutions attempt to develop policies and procedures for ensuring research integrity as required, adopt actions to set up academic committees, develop working plans for the investigation of research misconduct, and assign specific requirements for research integrity training. Although the national regulation sets forth such conditions, the operational procedures or standard guidelines are not further elaborated. Consequently, assessing whether the institutional level requirements developed are appropriate or at least satisfy the decent minimum requirement is difficult because of the absence of such criteria or guidance. Simultaneously, how to assure compliance is another concern.

Second, the quality issue is another common concern and has been widely discussed in research ethics reviews perennially, more recently, in the handling of research misconduct. This concern is closely related to the compliance issue mentioned above, which might radically affect the fundamental policy design of institutional responsibilities in research integrity in China. Many variations also exist among institutions, not only in tailored institutional policies but also in the capacity building of each institution. Such variations in capacities might have a far-reaching negative impact on the overall quality of institutional policies, even resulting in a double standard or mistrust among institutions, and finally, adversely affecting research integrity as a whole.

The third is the long-lasting problem of low efficiency. Efficiency concerns might have different aspects. More practically, take the ethics review as an example. The ethics review in China is mainly based on institutions. Usually, obtaining ethics approval for items reviewed by a convened board meeting may need approximately 2 months. More time is needed for multicenter research because of possible duplicated reviews. For research integrity investigation, the 2019 Rules allowed for 6 months at most to investigate alleged research misconduct; however, the practice always takes longer. A broader concern regarding efficiency is that each institution has to start over again to develop its institutional policies and procedures. This renders the institutional level policy development process time-consuming without a guarantee of quality. Consequently, considerable time, resources, and efforts are wasted.

## Discussion

The reasons for these identified challenges are complex. The compliance concern can be attributed to the vagueness and general characteristics of the regulatory requirement. A familiar voice was that the regulation requires an institution to set up a daily working mechanism to promote research integrity while failing to illustrate more details for practice. For instance, which department in the institution should bear such responsibilities? What are these daily working mechanisms? Thus, institutions may not know how to enforce this regulatory mandate. It may be comprehensible, but only to some extent. Evidently, given the nature of the regulations, it alone cannot answer these practical questions in detail. An accurate understanding and interpretation of the regulatory mandate play a crucial role during its application. Nevertheless, a huge gap still exists because, at least hitherto, no entity bears this burden. Top-down mechanisms do not yet exist to develop operational guidelines to support institutions. Meanwhile, the institutions seem to be passively waiting rather than creating institutional policies. Although many reasons may have led to this problem, failing to exercise discretion at the institutional level hindered the bottom-up strategy to mitigate this gap. More efforts are needed at various levels to identify possible solutions.

The quality issue is deeply rooted in the absence of standard criteria, which is worrisome. Consequently, institutions might hesitate to act before they have a concrete plan, which is a widespread phenomenon. Some institutions might be even afraid of possible criticism because of quality concerns. Although this perception is misleading, the main point is that concern about the quality issue should never be an excuse for taking no action. Stakeholders must be sensitized about this not being a suitable choice for fostering research integrity. Besides, the quality concern should be directly confronted and analyzed with more reasonable consideration and a practical action plan. Lack of evidence and evaluation scheme are the two main factors that need further examination. Thus, more proof and academic studies are required to establish standard criteria and evaluation metrics. As a starting point, some steps should be implemented to ensure institutions function well to fulfill their duties. For instance, they must identify detailed qualification requirements for research integrity management personnel and ethics review members, as well as personnel training requirements, investing resources to facilitate research integrity training, infrastructure development, and team building at the institutional level.

The compliance and quality concerns should be settled when dealing with efficiency. Whether a particular institution is ready to fulfill its responsibilities in promoting research integrity depends heavily on the institutional level operational policies, responsible personnel, infrastructure, institutional environment and culture, and most importantly, the cooperation of research personnel. All these factors vary considerably among different institutions, rendering the facilitation of research integrity development as a whole more complex. Communication and exchange among other institutions are still not standard. To some extent, the missing trust between institutions hindered possible efficiency. For instance, in duplicated ethics reviews of multicenter research, although mutual recognition of ethics review is encouraged by the regulation to enhance efficiency (2018), survey results showed that the willingness for such recognition is still low. Reasons include legal responsibilities of ethics review, quality variations, and accountability of research personnel management, among which, lack of trust between institutions was prominent (Tang, 2019).

## Practical proposal

Besides all these abovementioned concerns, another big challenge that is deeply hidden but has significantly impacted the development of research integrity in China is the openness and sharing of institutional experiences. Although a Chinese scholar developed a five-pronged strategy proposal to cultivate research integrity, which includes aligning norms, optimizing approaches, empowering enforcement, assigning responsibility, and enabling integrity (Yu et al., 2022), the proposal sets the framework, and in this perspective, we focus on more practical details at the institutional level to develop research integrity in China. To summarize, advocating that institutions should be more proactive and transparent in promoting research integrity is reasonable and necessary. The following practical proposal may help to justify possible action advocacies.

In the long term, institutions should reposition their roles in fostering research integrity. Along with recognizing the intrinsic value of research integrity for science and society, institutions and all other stakeholders should go beyond compliance and be more active and responsible in promoting research integrity. Institutions should adopt the following actions more proactively and responsibly with this common goal.

### Team building

Research integrity professionals and expert personnel should be cultivated and employed to assume the responsibility of managing and fostering research integrity at different levels. At the institutional level, a research integrity professional position should be established, and responsibilities, appropriate assessment and appraisal, career arrangement, and promotion path should be clarified to ensure the sustainable development of research integrity management. Research integrity professionals must be encouraged to conduct academic research or quality improvement activities. Simultaneously, institutions need to ensure the necessary resources and infrastructure to support these efforts.

### Policies innovation

Equipped with research integrity professionals, necessary resources, and infrastructure, institutions should be more confident in developing institutional working policies and procedures, adopting regulatory mandate as the bottom-line requirement, and, if possible, extending beyond to create more tailored and institution-specific research integrity development strategies. Brainstorming, expert consultation, and focus group discussions can be adopted as methodologies for institutional policy development. Voices and engagement of the research community, research integrity manager, ethics review members, as well as graduate students, if possible, are significant. Furthermore, internal quality assurance plans should be designed to monitor these policies and procedures to ensure they are user-friendly, effective, and efficient. Follow-up corrective activities and training should be monitored regularly.

### Capacity building

Besides team building and policy innovation, institutional capacity building is crucial for compliance with regulatory requirements, as well as for the sustainable development of research integrity. The minimum requirement for capacity building is ensuring all the personnel engaged in research integrity management are sufficiently competent to fulfill their responsibilities. Research integrity training, quality assurance plans, and quality improvement activities are helpful strategies for internal capacity building. Most importantly, continuous efforts should be undertaken to facilitate and maintain the capacity building.

### Researcher empowerment

Research personnel is the primary target audience for research integrity management. The engagement of the research community in institutional research integrity policymaking, training, and quality assurance is indispensable. The voice of research personnel should be heard at the outset of developing appropriate and tailored institutional research integrity policies. Research personnel should be fully informed about all relevant policies and procedures before applying. Feedback and suggestions from the targeted audience should be collected regularly for monitoring and possible quality improvement. Furthermore, research integrity consultation and training should be provided with easy access and convenience. At the same time, the education and research integrity training for students (both undergraduates and graduates) should be reinforced at institutional level to assure the preparation of next generation researchers.

### Experience sharing

Besides the institutional endeavors, each institution has a unique experience in fulfilling its responsibilities in developing research integrity, which is valuable and helpful. Therefore, institutions must be encouraged to openly share their lessons and experiences learned during their journey. Other peer institutions can benefit by learning from the shared experiences. Moreover, such experience sharing may reduce wastage at individual institutions that might otherwise restart developing research integrity. However, when advocating this, sharing culture and environment should be developed and nurtured. The uniqueness of each institution should be respected, tolerance for possible mistakes during the exploration of new policies should be accepted, and experiences shared should be credited. Furthermore, it is also necessary to develop a monitoring mechanism to propose warning or punishment timely when the institution failed to commit to promoting research integrity practically.

## Data availability statement

The datasets presented in this article are not readily available because, it is a perspective paper mainly illustrate the personal opinions. Requests to access the datasets should be directed to HZ, zhanghh@bjmu.edu.cn.

## Author contributions

The author confirms being the sole contributor of this work and has approved it for publication.

## Conflict of interest

The author declares that the research was conducted in the absence of any commercial or financial relationships that could be construed as a potential conflict of interest.

## Publisher's note

All claims expressed in this article are solely those of the authors and do not necessarily represent those of their affiliated organizations, or those of the publisher, the editors and the reviewers. Any product that may be evaluated in this article, or claim that may be made by its manufacturer, is not guaranteed or endorsed by the publisher.
